# Cephalic Version in the Postural Position

**Published:** 1882-02

**Authors:** John Hauenstein

**Affiliations:** President of the Erie County Medical Society


### BUFFALO

### Medical and Surgical Journal.

Vol. XXI. FEBRUARY, 1882. No. 7.

### Original Communications.

### Cephalic Version in the Postural Position.

ADDRESS OF DR. JOHN HAUENSTEIN, PRESIDENT OF THE ERIE COUNTY MEDICAL SOCIETY. READ AT THE ANNUAL MEETING, JANUARY 10, 1882.

*Gentlemen*—The few remarks which I intend to make, in an attempt to fulfill the requirements of an established usage, I commit on paper at a time when I contemplate, after a residence in this city for fifty years, the realization of a hope I long entertained, once more to see my native country, and to enjoy a few days of recreation and also of profit if opportunities present. Confident that the gentleman whose duty it will be to officiate as president in my absence, is well qualified for the position, and that the interest and welfare of the society will be represented by him with intelligence and prudence, the apology which I owe you for deserting the post of duty, need therefore but reflect my undeservedness of the honor you have conferred upon me. If my greetings to you are anticipated, I will repeat them in thought from afar, when my friend Dr. Bartow, whom I have delegated to present this paper at the proper time, will be granted the opportunity to read it. I sincerely hope that upon my return, I shall find the good feeling of fellowship of the members of this society unimpaired, and that not a single one be missing.

It is customary on occasions like this to treat upon some popular subject, so that not only the physician, but also the public in general, may profit by the labor and efforts of him, who so happily may have chosen his theme. Most of such subjects, however, in order to attract attention, must be treated by those who wield an abler pen than mine. I will therefore confine myself to narrower limits, and say, although not without diffidence, what I have to say, on the subject which I have chosen, to my colleagues, and if it have any merits it will alone interest them.

In the course of my obstetrical practice it has been my lot to meet with a number of face presentations, mostly in consultatation with the family physician, or to supersede a midwife in attendance; and ever since the postural treatment of the cord came into vogue, I have adopted a method in the management of face presentations, that invariably gave me satisfactory results. This method is *cephalic version* in the *postural position* or knee and chest position.

Authors on midwifery make no mention of the postural position in connection with the treatment of face presentations as far as I am aware, although I have searched quite a number of works; with but few exceptions, the modern accoucheurs do not favor cephalic version, but, on the contrary, there are some who denounce it most emphatically as a dangerous operation. There is no doubt in my mind that the condemnation of bringing down the vertex, regardless of posture, has arisen from the difficulty attending its execution, and the frequently complete failure of its accomplishment; and that, therefore, the older practitioners who thought active interference in face labors absolutely necessary, preferably performed podalic version.

Dr. Ramsbotham says: "Nor can we, indeed, succeed in producing the same alteration (to a vertex presentation) by the introduction of the hand over the vertex, the adaptation of the points of the fingers to the occiput, and the application of gentle traction, as some have recommended." "When it is possible to push the whole mass back," writes Dr. Meigs, "and bring down the vertex, let it be done, if deemed really necessary, but the opportunity to do this good action will rarely occur in practice."

Says Dr. Cazeaux: "I am now convinced that this manœuvre (bringing down the vertex) will rarely prove successful, therefore it should be attempted very carefully, and pelvic version substituted for it without much delay." Dr. Playfair in passing stricture on Dr. Hodge, who is one of the very few that think cephalic version, in all cases of face presentation, should be made, if called early, the presentation recognized, the os uteri dilated, and before the presenting part has passed this opening, says: "It may, however, be allowable in certain cases in which the face remains above the brim, and refuses to descend the pelvic cavity. Even then it is questionable whether podalic version should not be performed, as being easier of performance." Dr. Leishman, referring to cephalic version, makes the following objection: "It was therefore a totally erroneous impression of the nature of these labors which led Baudelocque to suggest, and so many of his followers to adopt, an operation which is scarcely less objectionable than turning." It is needless, however, to quote authorities on this point, since it is generally agreed by them that it is a difficult operation, and that it had better not be done, except under very favorable circumstances.

But the case is changed at once when the woman is placed in the knee and chest position; an operation which before was most difficult to execute, becomes now one of comparative ease. The head of the child, which before applied itself firmly to the brim of the pelvis, has now receded so that the hand can readily be passed-by it; or if it have already engaged in the upper strait, it can be made to recede by gentle pressure of the hand, all this in consequence of gravitation, the sinking of the gravid uterus forward and away from the brim, the cervix being no longer the most dependent portion.

But why attempt an operation of this kind, however easily performed, when writers on obstetric practice tell us that face presentations are natural labors, and that the female can expel the child with but little more difficulty in this case than in the vertex position; that if the child be not large, and the pelvis of the mother is well formed, little apprehension need be entertained by the accoucheur as to the result. Encouraging as are these words for the young physician, I venture to say that the practitioner who has met with a number of cases of face labor (particularly if in primiparas) in his practice, will hesitate before giving his endorsement to such views; and I am inclined to believe that such opinions are asserted and taught with some hesitation and distrust even by those who originate them.

Thus we find in the following statements made by authors, after having expressed opinions such as have been quoted, conclusive evidence that they consider face presentations occurrences of much more serious character than would permit them to be classed, unqualifiedly, among natural labors. Dr. Cazeaux says: "Nevertheless we must remark that, as a general rule, the labor is more tedious, more painful, and more dangerous, both to the mother and the child, and that it much oftener demands the intervention of art."

"We think, therefore," says Dr. Hodge, "that face presentations, although often accomplished with safety to the child and the mother, should be placed under the head of complicated labor; for in all cases there is necessarily a loss of power from the direction in which the bearing-down efforts operate, rendering labor tedious and painful to the mother. This tediousness is augmented whenever the head descends transversely, and especially when the os frontis is towards the anterior part of the pelvis; while in those cases where the os frontis comes directly to the pelvis, the dangers to the mother are excessively great.

Dr. Playfair remarks: "As regards the mother, in the great majority of cases the prognosis is favorable, but the labor is apt to be prolonged, and she is, therefore, more exposed to the risks attending tedious delivery. As regards the child, the prognosis is much more unfavorable than in vertex presentations. Even when the anterior rotation of the chin takes place in the natural way, it is estimated that one out of ten children is still-born; while if not, the death of the child is almost certain."

As to the appearance of the child after birth, Dr. Meigs says: "There can rarely be met with a more disagreeable spectacle than that of a new-born child's face, born after a bad face labor. It is frightfully suggillated, and often covered with blebs filled with yellow or bloody serum, the lips are completely extrophied, the eyes closed by infiltration of the palpebræ, and the nose enormously swollen." A face, let me add, suggestive of a brutal pugilistic encounter; the members of the mother's family pointing the finger of scorn at the physician in attendance as having had his fist in the fray.

Quotations could be multiplied *ad libitum*, if it were necessary farther to show that face labors are frequently the most unwelcomed occurrences in the practice of the accoucheur.

After a careful consideration of what has been said by writers on midwifery on face positions, and upon reviewing my own limited experience on the subject, I am forced to the conclusion, that cephalic version ought to be made in all cases, whenever it is practicable to do it without injury to the mother or child; and, furthermore, I am convinced that this operation can be performed in the *postural position* in a great many cases, where it were useless to attempt it should this precaution be neglected.

As regards the mode of procedure, you have, doubtless, anticipated the remarks that I intend to make on this part of my subject. The woman in labor is placed on her knees and chest, so that her trunk forms an inclined plane, the pelvis being the height and the knees and chest the base of the incline; and if unable herself to retain this posture, she is to be assisted and held in the required position by attendants. There are some who prefer resting the chest upon their hands, with their elbows extended sideward, in order to steady their bodies: in cases where we have reason to believe that version can easily be accomplished, the strictly knee and elbow position may be permitted, although, in most cases, it will not give the body the necessary degree of slope most favorable to insure success in the required manipulation.

The woman being thus placed in the *postural position*, and it having been ascertained whether the chin be to the left or to the right—accepting, as I shall, only the two fundamental positions of face presentations, viz: one with the chin toward the left, and one with the chin toward the right, as being sufficient for the practical purpose of cephalic version—that hand is introduced whose palmar surface embraces the vertex most readily, which will be the *right* hand when the chin is toward the *left* side, and *vice versa*. Pass the hand as far as to grasp the occiput with the four fingers, then flex the head on the chest, when the position is converted into one of the vertex. This procedure, however, is more easily described than accomplished, since the round ball-like surface now clapsed is lubricated with more or less sebaceous matter, causing the hold of the hand to easily slip; yet, by the persistent exertion of flexion and drawing upon the occiput, the effort will, after a short time be successful.

But it occasionally happens that the waters have broken many hours before the arrival of the physician, and the uterus has contracted somewhat firmly upon the child; the head, perhaps, is already engaged in the cavity of the pelvis to some extent. In such a case version becomes more difficult; and it may even be necessary in order to secure a firm hold, to impress the points of the fingers upon the child's scalp to effect the purpose—a procedure in the manipulation, that, in my opinion, can be conscientiously adopted, inasmuch as a few impressions or even abrasions on the scalp bear no comparison to the harm to a child's face, born after a face labor. Force, judiciously applied, is necessary in most obstetric operations; and, although it may shock the sensitiveness of some highly susceptible person, the accoucheur is nevertheless justified in the use of considerable force to convert a face position into one of the vertex.

When the face has passed the superior strait and is lodged in the cavity of the pelvis, where its farther progress is interrupted, either by uterine inertia, or by some abnormal impediment, giving rise to non-rotation of the chin forward, various methods are recommended in books to correct the difficulty. Among these may be mentioned, the introduction of the finger into the mouth of the child and drawing the chin forward during a pain; again, the passing of the finger up behind the occiput and pressing it backward during the pain. Penrose, in the belief that non-rotation is generally caused by the want of a *point d'appui* below, applies the hand, or the blade of the forceps, so as to press on the posterior cheek. If, however, rotation does not take place, notwithstanding these devices, then podalic version is advised to be tried; next in order comes the vectis, after which the forceps are recommended to effect rotation, and, if unsuccessful, an attempt is to be made with them to draw the face downward. Finally, if all these measures should fail, the operation of craniotomy is a last expedient.

Such are the resources, which I find mentioned in obstetric works, and from which we are to select the particular method most applicable in the case on hand, in preference to an attempt at dislodging the head from the pelvic cavity and bringing down the vertex.

The following condensed report of a case of difficult face labor, however, would almost imply criminal neglect of the means at our command in desperate cases, if an attempt at reduction and version of the head had not been made. Of posture nothing is said, and I therefore take it for granted that the operation was made without its advantage. Dr. Jno. S. Parry, the editor of the American editon of "Leishman's System of Midwifery," in a paper read before the Obstetric Society of Philadelphia, reports: "In this (the paper) was related a case of face presentation, with the chin behind and to the right side, seen in consultation with Dr. Elliot Richardson. The face was almost at the inferior strait. All attempts to flex the head, to rotate the chin in front, or to deliver by traction with the forceps failed. The woman was completely exhausted, and there seemed to be no alternative but to perform craniotomy. Before resorting to this, however, I passed my whole hand into the pelvis, and placing the thumb over the brow and the fingers over the superior maxillary bone, and pushing forcibly upward the head was easily raised above the brim of the pelvis. It was then flexed without any difficulty, and a mento-posterior of the face was converted into an occipito-anterior of the vertex. Wallace's forceps were promptly applied, and a few minutes later we had the satisfaction of delivering a living child."

If then, in so desperate a case as the one cited, reposition and version of the head could be accomplished in the manner heretofore practiced, I have strong reason to believe that a case would have to be a very hopeless one if it could not be corrected with the additional advantage of posture. Yet, no one will deny but that cases will occur when all efforts to dislodge the head from the pelvic cavity and to force it above the brim will fail.

I will now relate a few cases which happened within less than two years, to which I was called in consultation by a very intelligent physician,—one who has a large practice in midwifery. I might report several cases that happened years ago, but having kept no record of them will content myself by reporting briefly, but three cases, the facts in which can be corroborated by my friend Dr. Ring. That the three cases should have occurred in the practice of one physician, within the short period of less than two years, may appear somewhat unusual; but coincidences are not infrequent in connection with the other accidents to which the parturient is liable,—the accoucheur may meet with a number of cases of eclampsia, placenta prævia, &c., within a few weeks or months, and then, perhaps, see no more of such cases for several years.

*Case I.* Mrs. Van V., aged 31 years, tall, with well marked lateral curvature of the spine, was taken with labor pains with her first child at 9 o'clock P. M. on the twentieth of June 1879, and in the absence of Dr. W. Ring, his son, Dr. Chas. A. Ring, attended her during the night. In the morning, Dr. W. Ring called and found the os uteri dilated to the size of a half dollar, the face presenting with the chin toward the left side. At 10 o'clock the following morning I first saw the patient in consultation with Drs. W. and Chas. A. Ring. An examination confirmed their diagnosis, and detected at the same time a marked projection of the sacrum forward, and consequent diminution of the conjugate diameter of the pelvis. The membranes had ruptured and the face was pressed firmly against the brim. It was agreed to attempt version of the head. The woman was placed in the knee and chest position, and I then passed my right hand into the uterus as far as enabled me to grasp the occiput, then flexing the head upon the chest, I succeeded in bringing down the vertex. In consequence, however, of the deformity, the head did not engage in the pelvis, and three hours after the version of the head the forceps were applied and the woman delivered of a healthy child. The mother's convalescence was natural, and the child was thrifty in appearance.

*Case II.* Dr. W. Ring sent for me at 10 o'clock A. M., December 8, 1880, to assist him in a case of face labor. The woman, Mrs. S., 29 years of age, with her first child, had been in labor twenty-four hours before I saw her. An examination proved the chin to be to the left side, and not yet engaged in the upper strait. In consultation it was agreed, in order to expedite the labor and to relieve the woman of her sufferings, to resort to cephalic version. She was placed in the postural position, and my right hand was then passed over the occiput, and the face presentation was converted into one of the vertex. Labor was completed by the natural powers two hours after version. Mother and child made a good recovery, although the mother needed more than ordinary care to guard against inflammation.

*Case III.* Mrs. C., aged 35 years, fleshy and strong, commenced her fourth labor at 5 o'clock A. M., March 17, 1881.

Dr. Ring was called at 8 o'clock, and found membranes ruptured, the child presenting face downward, chin toward the left sacro-iliac symphysis but more nearly to the sacrum, child's head very large. At 10 o'clock A. M., Dr. Ring sent for me, and upon my arrival I found the condition as stated. The woman had powerful labor pains, and the face of the child was pressed with great violence against the brim of the pelvis, causing it to swell considerably. As soon, however, as the woman was placed in the postural position, my hand could readily be passed into the uterus; I found the fœtal head to be free and very movable, so much so that in order to fix it, I had to grasp the occiput with the points of my fingers. Version being completed, the child was born twenty minutes after; the effects of pressure were shown by a swelled face and closed eyes for several days, consequent upon the strong labor pains. The child weighed twelve pounds. Mother and child made an excellent recovery.

Such being some of my experience in face presentations, I concluded, after some hesitation, to acquaint my professional colleagues of this society with the facts of a mode of procedure in such cases, that I have not found mentioned in the writings of obstetricians. For any merit that there may be in this method of correcting face presentations, I can truthfully say I am under obligation to no one for hints or suggestions in its adoption. The claim for originality, however, is immaterial; if I have succeeded in calling your attention to a practice which you will recognize and acknowledge after trial as an improvement in the management of face labors, *my labors* are compensated.

